# Association of *COL4A1* genetic polymorphisms with coronary artery disease in Uygur population in Xinjiang, China

**DOI:** 10.1186/1476-511X-12-153

**Published:** 2013-10-25

**Authors:** Dilare Adi, Xiang Xie, Yi-Tong Ma, Zhen-Yan Fu, Yi-Ning Yang, Xiao-Mei Li, Yang Xiang, Fen Liu, Bang-Dang Chen

**Affiliations:** 1 Department of Cardiology, First Affiliated Hospital of Xinjiang Medical University, Urumqi 830054, People’s Republic of China; 2 Xinjiang Key Laboratory of Cardiovascular Disease Research, Urumqi, 830054, People’s Republic of China

**Keywords:** COL4A1, Single nucleotide polymorphism, Coronary artery disease, Case–control study

## Abstract

**Background:**

Type IV collagen is important for the structural integrity and function of basement membranes. Basement membranes surround vascular smooth muscle cells in the media, COL4A1 is the most abundant component of type IV collagen in all Basement membranes. However, the relationship between COL4A1 genetic polymorphisms and coronary artery disease (CAD) remains unclear. We performed a case–control study to explore the association of COL4A1 genetic polymorphisms with CAD in Uygur population of China.

**Methods:**

1095 Uygur people (727 men, 368 women) including 471 CAD patients and 624 controls were selected for the present study. Two SNPs (rs605143 and rs565470) were genotyped by using the polymerase chain reaction-restriction fragment length (PCR-RFLP) method.

**Results:**

For total and men, the rs605143 was found to be associated with CAD by in a dominate model (p = 0.014, p = 0.013, respectively). The difference remained statistically significant after multivariate adjustment (p = 0.036, p = 0.014, respectively). The rs565470 was also found to be associated with CAD in a recessive model for total and men (both p < 0.001), and the difference remained statistically significant after multivariate adjustment (P = 0.002, P = 0.001, respectively).

**Conclusion:**

Both rs605143 and rs565470 of COL4A1gene are associated with CAD in Uygur population of China.

## Introduction

Coronary artery disease (CAD) is the leading cause of death worldwide, which is caused by multiple interacting endogenous and exogenous factors. In recent years, genetic basis of CAD has gained considerable interest [1]; heritable factors accounted for 40%-60% in occurrence and development of CAD [2]. Genome- wide association studies (GWAS) some large-scale association analysis have identified many common, uncommon and functional variants for CAD [3,4].

Collagens are a group of proteins characterized by a unique sequence whose every third amino acid is a glycine (Gly-Xaa-Yaa motif). There are at least 25 different types of collagens. The most abundant four found in mammals are Type I, II, III and IV collagens [5]. Among them, Type IV collagen is the main component comprising 50% of all basement membrane (BM) and is expressed at all tissues including the vasculature, renal glomerular and ocular structures [6,7]. The vascular BM is an important component of the vasculature. BM surrounds vascular smooth muscle cells in the media making up the barrel of every blood vessel and capillary [8]. As is known, integrity and stability of blood vessels are critical to vascular system, and maintenance of vessel system can be successfully achieved by cooperation of many constituents of blood vessels such as endothelial cells, pericytes, and BM. However failure of this system results in serious consequences such as hemorrhage, edema, inflammation, and tissue ischemia. Type IV collagen, a major component of vascular BM, could provide physical barrier to both soluble molecules and migrating cells and could function as scaffold that allows interaction between pericytes and endothelial cells, thereby contributing to vessel stabilization and enables BM to with-stand mechanical stress [9].

COL4A1 gene is mapped on the telomeric region of 13q (13q34) and consists of 52 exons encodes a1 chain of type IV collagen [10]. In recent years, understanding of polymorphisms of the COL4A1 gene reached a certain degree. For example, Yamada Y et al. [11] identified a novel polymorphism in the COLA1 which is significantly associated with prevalence of myocardial infarction. A Genome-Wide Association Scan (GWAS ) study conducted by Kirill V et al. reported that COL4A1 gene is associated with arterial stiffness [12]. However, the relationship between polymorphisms of COL4A1 gene and CAD remains unclear.

In the present study, we aimed to clarify the association of COL4A1 gene polymorphisms with CAD in a Uygur population of China.

## Methods

### Ethical approval of the study protocol

This study was approved by the Ethics Committee of the First Affiliated Hospital of Xinjiang Medical University (Xinjiang, China). It was conducted according to the standards of the Declaration of Helsinki. Each participant gave written informed consent and explicitly provided permission for DNA analyses as well as collection of relevant clinical data.

### Study population

Study population is comprised of 1095 Uygur people (726 men, 368 women) who lived in Xinjiang Uygur Autonomous Region of China. Among them, 471 subjects attended as inpatients at the First Affiliated Hospital of Xinjiang Medical University between January 2006 to 2011 and underwent coronary angiography, diagnosed with CAD (based on the presence of at least one significant coronary artery stenosis of > 50% luminal diameter). Data and information about traditional coronary risk factors (including hypertension, diabetes mellitus, and smoking) and other biochemical indices were collected from all participants. The diagnosis of hypertension was established if patients were on anti-hypertensive medication or if the mean of 3 measurements of systolic blood pressure (SBP) ≥ 140 mmHg and/or diastolic blood pressure (DBP) ≥ 90 mmHg. Diabetes mellitus was defined on the basis of the World Health Organization (WHO) criteria. Smoking was classified as smokers (including current or ex-smokers) or non smokers. Patients with congenital heart disease, multiple organ failure syndrome and drug users were excluded from this study.

The control subjects were comprised of 624 subjects selected from the Cardiovascular Risk Survey (CRS) [13,14]. This Survey consists of 14,618 subjects and is a multiple-ethnic, community-based, cross-sectional study designed to investigate the prevalence, incidence, and risk factors for cardiovascular diseases in Han, Uygur, and Kazakh population in Xinjiang (west China) between June 2007 and March 2010. Individuals who had underwent heart bypass surgery, coronary stenting, had history of myocardial infarction, CAD, electrocardiographic signs of CAD, and relevant valvular abnormalities in echocardiograms were excluded from this group.

### Biochemical analyses

5 mL of fasting venous blood was collected into tubes containing ethylene diamine tetraacetic acid (EDTA). The collected samples were centrifugated into plasma, serum and blood cells (including leukocytes). Genomic DNA was extracted from the peripheral leukocytes using standard phenol-chloroform method and stored at -80°C for future analysis. We used standard methods for chemical analysis(Dimension AR/AVL Clinical Chemistry System, Newark, NJ, USA) employed by the Clinical Laboratory Department of the First Affiliated Hospital of Xinjiang Medical University [15,16]. Serum concentrations of total cholesterol (TC), triglycerides (TG), glucose, high-density lipoprotein cholesterol (HDL-C), low-density lipoprotein cholesterol (LDL-C), blood urea nitrogen (BUN), creatinine (Cr) and uric acid were measured.

### Genotyping of COL4A1 gene

**Figure 1:**
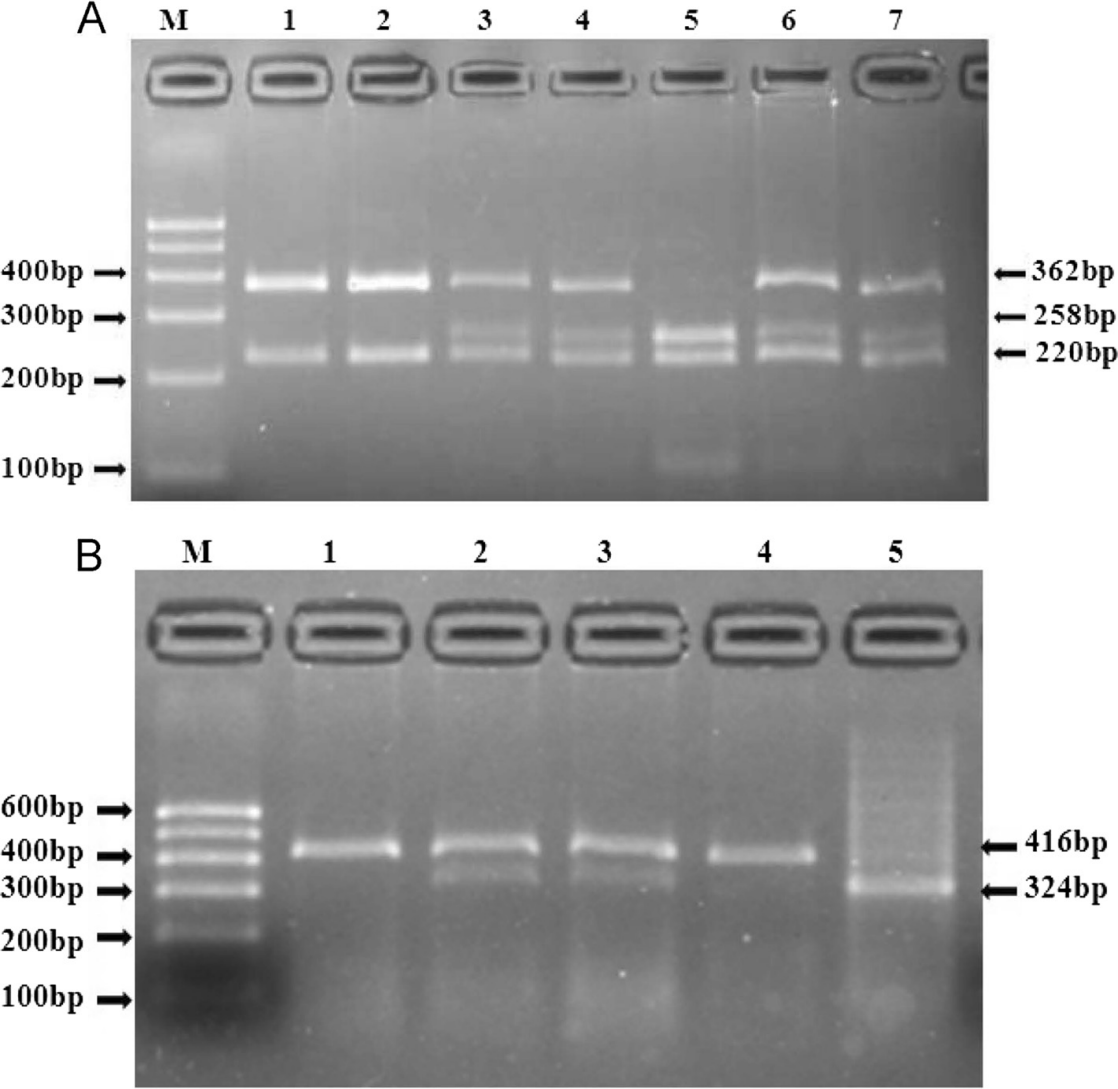
**Restriction fragment length polymorphism analysis for determination of genotype. A**. For SNP1, the GG genotype shows two bands of 362 bp and 220 bp (1 and 2); The AG genotype shows three bands of 362 bp, 258 bp and 220 bp (3, 4, 6 and 7), The AA genotype shows three bands of 258 bp, 220 bp and 104bp (5). **B**. For SNP2, the TT genotype shows one band of 416 bp (1 and 4); The CT genotype shows two bands of 416 bp and 324 bp (2 and 3); The CC genotype shows one band of 324 bp (5).

Using Haploview 4.2 software and International HapMap Project website phase I &II data base (http://www.hapmap.org), we obtained two tag SNPs: SNP1 (rs605143) and SNP2 (rs565470) by using minor allele frequency (MAF) ≥ 0.05 and linkage disequilibrium patterns with r^2^ ≥ 0.8 as a cutoff. Genotyping in this present case–control study was confirmed by polymerase chain reaction (PCR)-restriction fragment length polymorphism (RFLP) analysis. Sequence information for use as a reference template was obtained from the Ensembl Genome Browser (Human, numberENSG00000187498). Sequencing primers were designed using Primer Premier 5.0 software, synthesis of the Premier was undertaken by Shanghai Genery Biological Technology Company Limited (Shanghai, China). PCR amplification was performed using 25 uL of 2*powder Taq PCR master mix (Beijing Biotech, Beijing, China), 50 ng of genomic DNA, 21 uL of distilled water, 1uL of each forward and reverse primer in a 50 μL final reaction volume. The thermal cycling conditions were as follows: an initial denaturation step at 95°C for 5 min; 30 cycles of 95°C for 30s, 60°C for 35 s and 72°C for 1 min was followed by a final extension step of 72°C for 10 min. Thermal cycling was performed using the GeneAmp 9700 system (Applied Biosystems) and. PCR products were digested by restriction enzyme (Fermentas, Beijing, China) in a 20 μL final reaction volume, along with 10 μL of PCR product, 5U of restriction enzyme, 9 uL of distilled water and 1uL Solution Buffer, incubated overnight at 37°C. The primer pair sequences, annealing temperatures, resulting fragments and restriction enzymes for the two SNPs are detailed in Table 1. Resulting fragments were separated on 3.0% agarose gel (Figure 1). Finally to ensure the results to be verified, we used sequenced genomic DNAs as positive controls in our assays.

**Table 1:** Primer sequences of each SNP

**SNPs**	**Polymerase chain reaction primers**	**Denaturation temperature**	**Products length**	**Restriction enzyme**
rs605143	Sense 5′AAAGCCATTGCTACCTCA3′	60°C	582 bp	DraI
	Antisense 5′CTGCTCCTGGTGACTCTG3′			
rs565470	Sense 5′GAATGCGATAAGGACAGGG3	60°C	416 bp	BanII
	Antisense 5′AGGAAAGGGAGGCACAAAA3′			

### Statistical analyses

All continuous variables were expressed as mean ± standard deviation (S.D), and the differences between CAD and control group were analyzed by using an independent-sample t-test. Differences in frequencies of smoking, drinking, hypertension, diabetes mellitus, and COL4A1 genotypes were analyzed using χ^2^ test or Fisher’s exact test while appropriate. Hardy–Weinberg equilibrium was assessed by χ^2^ analysis. Logistic regression analyses with effect ratios (odds ratio [OR] and 95% CI) were used to assess contribution of major risk factors. P value < 0.05 was considered statistically significant. All statistical analyses were performed by using SPSS17.0 software for Windows (SPSS Institute, Chicago, USA).

## Result

Table 2 shows demographic and clinical characteristics of 1095 study subjects. For total, including men and women, BMI, systolic blood pressure, diastolic blood pressure, plasma concentration of glucose, triglyceride, uric acid and prevalence of conventional risk factors for CAD including hypertension and diabetes mellitus were significantly higher in subjects with CAD than in the controls, whereas plasma concentration of HDL cholesterol was higher in controls than in subjects with CAD. The plasma concentration of LDL cholesterol, total cholesterol were significantly higher in controls than in subjects with CAD. For woman, the BUN concentration was significantly higher in subjects with CAD than in controls. No significant differences were found in the following variables between CAD subjects and controls: age, drinking and plasma concentration of creatinine (Cr).

**Table 2:** Demographic and clinical characteristics of study participants

	**Total**			**Men**			**Women**		
	**CAD**	**Control**	**P value**	**CAD**	**Control**	**P value**	**CAD**	**Control**	**P value**
Number(n)	471	624		327	400		144	224	
Age, mean (SD)	56.07 (8.50)	55.95 (8.35)	0.825	55.30 (8.54)	55.77 (8.46)	0.46	57.81 (8.12)	56.28 (8.17)	0.082
EH, n (%)	247 (52.4)	199 (31.9)	< 0.001	159 (48.6)	129 (32.3)	< 0.001	88 (61.6)	70 (31.3)	< 0.001
Diabetes, n (%)	99 (21.0)	20 (3.2)	< 0.001	65 (19.9)	20 (5.0)	< 0.001	34 (23.6)	0	< 0.001
Smoking, n (%)	178 (37.8)	178 (28.50)	0.001	178 (54.4)	177 (43.3)	0.007	0	1 (4%)	1
Drinking, n (%)	70 (14.9)	80 (12.8)	0.375	70 (21.4)	79 (19.8)	0.644	0	1 (4%)	1
BMI, mean (SD)	27.34 (3.69)	25.24 (4.32)	< 0.001	27.47 (3.66)	25.23 (3.94)	< 0.001	27.06 (3.75)	25.26 (4.93)	< 0.001
SBP, mean (SD)	141.13 (29.32)	129.96 (19.57)	< 0.001	138.80 (29.31)	130.41 (19.56)	< 0.001	146.43 (28.74)	129.16 (19.61)	< 0.001
DBP, mean (SD)	87.74 (19.27)	80.03 (13.93)	< 0.001	86.74 (19.38)	80.57 (14.14)	< 0.001	89.59 (18.92)	79.08 (13.52)	< 0.001
Glu, mean (SD)	6.10 (2.41)	4.83 (1.35)	< 0.001	6.05 (2.38)	4.87 (1.58)	< 0.001	6.22 (2.50)	4.77 (0.79)	< 0.001
TG, mean (SD)	1.10 (1.16)	1.58 (1.08)	< 0.001	1.96 (1.16)	1.57 (1.12)	< 0.001	2.08 (1.15)	1.59 (1.02)	< 0.001
TC, mean (SD)	4.20 (1.10)	4.38 (1.06)	0.008	4.08 (0.98)	4.28 (1.01)	0.009	4.47 (1.28)	4.55 (1.11)	0.548
HDL, mean (SD)	0.94 (0.55)	1.26 (0.42)	< 0.001	0.91 (0.52)	1.28 (0.43)	< 0.001	1.01 (0.59)	1.25 (0.41)	< 0.001
LDL, mean (SD)	2.60 (1.00)	2.88 (0.97)	< 0.001	2.55 (0.99)	2.85 (0.95)	< 0.001	2.73 (1.03)	2.91 (1.01)	0.086
UA, mean (SD)	311.92 (86.07)	261.58 (73.79)	< 0.001	324.71 (81.03)	279.23 (72.68)	< 0.001	283.00 (90.50)	230.08 (64.84)	< 0.001
Cr, mean (SD)	78.77 (33.05)	78.34 (33.68)	0.833	83.69 (33.87)	85.98 (36.67)	0.385	67.58 (28.17)	64.68 (21.68)	0.226
BUN, mean (SD)	5.54 (2.29)	5.37 (1.92)	0.182	5.54 (2.37)	5.52 (2.07)	0.94	5.54 (2.11)	5.09 (1.59)	0.02

Continuous variables are expressed as mean ± SD. Categorical variables are expressed as percentages.

BMI, body mass index; BUN, blood urea nitrogen; Cr, creatinine; DBP, diastolic blood pressure; DM, diabetes mellitus; Glu, glucose; TG, triglyceride; TC, total cholesterol; HDL, high density lipoprotein; LDL, low density lipoprotein; EH, essential hypertension; SBP, systolic blood pressure; UA, uric acid;

The P value of the continuous variables was calculated by the Independent t-test.

The P value of the categorical variables was calculated by Fisher’s exact test.

Table 3 shows distribution of genotypes and alleles of SNPs for COL4A1 gene. The genotype distributions for each of the SNPs were in good agreement with the predicted Hardy – Weinberg equilibrium values (data not shown). For total, distribution of rs605143 genotypes, dominant model (GG vs AA + AG), recessive model (AA vs GG + AG) and allele frequency showed significant difference between CAD and control subjects (p = 0.02, p = 0.014, p = 0.048 and p = 0.005). For men, distribution of rs605143 genotypes, dominant model (GG vs AA + AG) and allele frequency showed difference between CAD and control subjects (p = 0.03, p = 0.013 and p < 0.001). For total and men, A allele of rs605143 was significantly higher in controls than in subjects with CAD (total: 46.2%vs 40.1%; men: 47.2%vs 40.4%), the dominant model (GG vs AA + AG) of rs605143 was significantly higher in subjects with CAD than in controls (total: 37.2% vs 30.0%; man: 37.0%vs 28.2%). For total, the recessive model (AA vs GG + AG) of rs605143 was significantly lower in CAD subjects than in controls (17.4%vs 22.4%).There was no significant difference between CAD and control subjects in women for distribution of rs605143 genotypes, dominant model (GG vs AA + AG), recessive model (AA vs GG + AG) and allelic distribution (p > 0.05).

**Table 3:** Genotype and Allele distributions in patients with CAD and control participants

	**Total**			**Man**			**Woman**		
**Variants**	**CAD n (%)**	**Control n (%)**	**P value**	**CAD n (%)**	**Control n (%)**	**P value**	**CAD n (%)**	**Control n (%)**	**P value**
rs605143 (SNP1)									
genotype									
G/G	175 (37.2)	187 (30.0)		121 (37)	113 (28.2)		54 (37.5)	74 (33.0)	
A/G	214 (45.4)	297 (47.6)		148 (45.3)	196 (49.0)		66 (45.8)	101 (45.1)	
A/A	82 (17.4)	140 (22.4)	0.02	58 (17.7)	91 (22.8)	0.03	24 (16.7)	49 (21.9)	0.424
Dominant model									
GG	175 (37.2)	187 (30.0)		121 (37)	113 (28.2)		54 (37.5)	74 (33.0)	
AG + AA	296(62.8)	437 (70)	0.014	206 (63.0)	287 (71.8)	0.013	90 (62.5)	150 (67.0)	0.433
Recessive model									
AA	82 (17.4)	140 (22.4)		58 (17.7)	91 (22.8)		24 (16.7)	49 (21.9)	
AG + GG	389 (82.6)	484 (77.6)	0.048	269 (82.3)	309 (77.3)	0.098	120 (83.4)	175 (78.1)	0.232
Additive model									
AG	214 (45.4)	297 (47.6)		148 (45.3)	196 (49.0)		66 (45.8)	101 (45.1)	
GG + AA	257 (54.6)	327 (52.4)	0.501	179 (54.7)	204 (51.0)	0.332	78 (54.2)	123 (54.9)	0.915
Allele									
G	564 (59.9)	671 (53.8)		390 (59.6)	422 (52.8)		174 (60.4)	199 (44.4)	
A	378 (40.1)	577 (46.2)	0.005	264 (40.4)	378 (47.2)	< 0.001	114 (39.6)	249 (55.6)	0.222
rs565470(SNP2)									
genotype									
T/T	169 (35.9)	308 (49.4)		104 (31.8)	192 (48.0)		65 (45.1)	116 (51.8)	
C/T	221 (46.9)	271 (43.4)		153 (46.8)	181 (45.2)		68 (47.2)	90 (40.2)	
C/C	81 (17.2)	45 (7.2)	< 0.001	70 (21.4)	27 (6.8)	< 0.001	11 (7.6)	18 (8.0)	0.392
Dominant model									
TT	169 (35.90)	308 (49.4)		104 (31.8)	192 (48.0)		65 (45.1)	116 (51.8)	
CC + CT	302 (64.1)	316 (50.6)	< 0.001	223 (68.2)	208 (52.0)	< 0.001	79 (54.9)	108 (48.2)	0.24
Recessive model									
CC	81 (17.20)	45 (7.2)		70 (21.4)	27 (6.8)		11 (7.6)	18 (8.0)	
TT + CT	390 (82.8)	579 (92.8)	< 0.001	257 (78.6)	373 (93.2)	< 0.001	133 (92.4)	206 (92.0)	1
Additive model									
CT	221 (46.9)	271 (43.4)		153 (46.8)	181 (45.2)		68 (47.2)	90 (40.2)	
TT + CC	250 (53.1)	353 (56.6)	0.269	174 (53.2)	219 (54.8)	0.709	76 (52.8)	134 (59.8)	0.196
Allele									
T	559 (59.3)	887 (71.1)		361 (55.2)	565 (70.6)		198 (68.8)	322 (71.9)	
C	383 (40.7)	361 (28.9)	< 0.001	293 (44.8)	235 (29.4)	< 0.001	90 (31.2)	126 (28.1)	0.407

CAD, Coronary artery disease; N, number of participants; SNP, single-nucleotide polymorphism.

For total and men, distribution of rs565470 genotypes, dominant model (TT vs CC + CT), recessive model (CC vs TT + CT) and allele frequency showed difference between CAD and control subjects (all P < 0.001). C allele of rs605143 was significantly higher in CAD patients than in control groups (total: 40.7% vs 28.9%; men: 44.8% vs 29.4%), the dominant model (TT vs CC + CT) of rs565470 was significantly higher in controls than in CAD patients (total: 49.4% vs 35.90%; men: 48.0% vs31.8%). The recessive model (CC vs TT + CT) of rs565470 was significantly lower in CAD subjects than in controls (total: 82.8% vs 92.8%; men: 78.6% vs 93.3). There was no significant difference between CAD and control subjects in women for distribution of rs565470 genotypes, dominant model (TT vs CC + CT), recessive model (CC vs TT + CT) and allelic distribution (p > 0.05).

Table 4 shows multivariable logistic regression analysis combining genotypes with following variables: plasma concentration of TG, TC, HDL, LDL, incidence of hypertension, diabetes and smoking which were the major confounding factors for CAD. For total and men (Table 4), after multivariate adjustment, rs605143 remain significantly associated with CAD in dominant model (for total: OR = 1.369, 95% confidence interval [CI]: 1.021-1.834, p = 0.036; for men: OR = 1.583,95% confidence interval [CI]: 1.099-2.281, p = 0.014). For total, after multivariate adjustment, rs605143 remains significantly associated with CAD (OR = 0.661, 95% confidence interval [CI]: 0.465-0.942, p = 0.022) in recessive model (data not shown).

**Table 4:** Multiple logistic regression analysis for CAD patients and control subjects (rs605143)

	**Total**			**Men**			**Woman**		
	**OR**	**95% CI**	**P**	**OR**	**95% CI**	**P**	**OR**	**95% CI**	**P**
Dominant model (GG vs AA + AG)	1.369	1.021-1.834	0.036	1.583	1.099-2.281	0.014	1.069	0.636-1.799	0.8
Hypertension	2.192	1.655-2.903	< 0.001	1.889	1.334-2.675	< 0.001	3.041	1.834-5.041	< 0.001
Diabetes	6.034	3.676-10.473	< 0.001	3.229	1.771-5.887	< 0.001	0	0	0
Smoking	1.381	1.029-1.854	0.032	1.422	1.009-2.005	0.044	0	0	1
TG	1.222	1.153-1.516	< 0.001	1.274	1.078-1.507	0.005	1.543	1.211-1.967	< 0.001
TC	0.78	0.669-0.908	0.001	0.744	0.612-0.904	0.003	0.78	0.595-1.022	0.072
HDL	0.15	0.097-2.30	< 0.001	0.1	0.058-0.171	< 0.001	0.406	0.197-0.840	0.015
LDL	0.949	0.811-1.111	0.0515	0.961	0.795-1.163	0.685	0.831	0.609-1.134	0.243

For total and men (Table 5), after multivariate adjustment, rs565470 remain significantly associated with CAD in recessive model (for total: OR = 1.993, 95% confidence interval [CI]: 1.277-3.112, p = 0.002; for men: OR = 2.506, 95% confidence interval [CI]: 1.475-4.258, p = 0.001) and in dominant model (for total: OR = 0.708, 95% confidence interval [CI]: 1.021-1.834, p = 0.016; for men: OR = 0.616, 95% confidence interval [CI]: 0.434-0.873, p = 0.006; data not shown for dominant model).

**Table 5:** Multiple logistic regression analysis for CAD patients and control subjects (rs565470)

	**Total**			**Men**			**Woman**		
	**OR**	**95% CI**	**P**	**OR**	**95% CI**	**P**	**OR**	**95% CI**	**P**
Recessive model (CC vs TT + CT)	1.993	1.277-3.112	0.002	2.506	1.475-4.258	0.001	1.055	0.412-2.705	0.911
Hypertension	2.156	1.627-2.857	< 0.001	1.847	1.304-2.618	0.0006	3.039	1.833-5.040	< 0.001
Diabetes	5.873	3.397-10.153	< 0.001	3.101	1.705-5.637	< 0.001	0	0	0.997
Smoking	1.285	0.956-1.726	0.096	1.297	0.921-1.827	0.136	0	0	1.000
TG	1.330	1.158-1.527	< 0.001	1.275	1.076-1.511	0.0051	1.545	1.211-1.970	< 0.001
TC	0.776	0.666-0.904	0.001	0.746	0.613-0.909	0.0036	0.778	0.593-1.019	0.069
HDL	0.151	0.098-0.233	< 0.001	0.104	0.060-0.179	< 0.001	0.407	0.197-0.840	0.015
LDL	0.964	0.824-1.128	0.645	0.982	0.812-1.188	0.853	0.832	0.610-1.135	0.246

## Discussion

We found that variation in COL4A1 gene is associated with CAD in a Uygur population of China. After multivariate adjustment, the associations between COL4A1 gene polymorphisms with CAD were not modified. This was the first study to investigate the common allelic variants in COL4A1 gene and its association with CAD in Uygur population of China.

Type IV Collagen was derived from glomerular basement membrane by Kefalides in 1966 [17]. Collagen type IV is major structural component of BM. Thus it is important for integrity and functions of BM [10]. There are 6 different types of type IV collagen α-chains (α1-α6). Each of the six chains of collagen IV has three domains: There is a short 7S domain at the N-terminal; along with collagenous domain occupying the midsection of the molecule, which contains the classic Gly-Xaa-Yaa repeated amino acid sequence and a non-collagenous domain (NC1) is positioned at the C-terminal. These six different collagen type IV alpha chains (α1- α6), form only three sets of triple helical molecules called protomers, which are designated as α1.α1.α2, α3.α4.α4, α5.α5.α6 [4,18-20], among them α1.α1.α2 is very crucial for the protein.

COL4A1 gene which encodes α1 chain of type IV collagen is a new gene identified in the CARDIoGRAM Consortium [4]. Mutations of COL4A1 gene have been initially reported in a mouse bearing a heterozygous that results in in-frame deletion of exon 40, this mutation causes abnormal synthesis of the protein and the protein cannot be properly secreted outside the cell, finally leading mouse prone to brain hemorrhage at their birth [21].In addition, Gould DB et al. [22] also observed that with in-frame deletion of exon 40 may predispose adult mouse to vascular fragility and act in concert with birth trauma to cause cerebral hemorrhage. The first description of a pathologic COL4A1 mutation in humans was reported in 2005, Gould and co-workers indicated that mutation in COL4A1 was related to congenital autosomal dominant porencephaly [21]. Since then, several researchers reported that mutation in COL4A1 gene results in human hemorrhagic stroke [22,23]. Besides, mutant mice also showed very tortuous retinal vasculature, perinatal haemorrhage, porencephaly and these conditions were also seen in a French family with familial infantile hemiparesis. Cassandre Labelle-Dumais et al. [24] demonstrates that mutations in COL4A1 gene are a major cause of Muscle-eye-brain disease (MEB) and Walker Warburg Syndrome (WWS), characterized by ocular dysgenesis, neuronal migration defects, and congenital muscular dystrophy, this finding could develop an assay to test the functional significance of putative COL4A1 mutations.

Research about the relationship between polymorphisms of COL4A1 gene and cardiovascular diseases was first reported by Yamada Y et al. [12], they demonstrated that the A → C (Gln1334His) polymorphism (rs3742207) of COL4A1 is associated with prevalence of MI in Japanese people, with C allele protecting against this condition. However, the underlying molecular mechanism is not clear. Tom Van Agtmael et al. [25] indicated that animals with a COL4A1 missense mutation resulting in a complex vascular phenotype including defects in maintenance of vascular tone, endothelial cell function and blood pressure regulation. One genome-wide association study (GWAS) for vascular stiffness measures reported a strong replicated association of SNP (rs3742207) in COL4A1 with arterial stiffness, this study suggesting previously unrecognized cell-matrix interactions may exert an important role in regulating arterial stiffness, but the mechanism to regulate arterial stiffness is not yet clear; further work is needed to elucidate these mechanisms [12].

In our study, we found that polymorphisms of COL4A1 were associated with risk of CAD in a Uygur population. There was significant difference in genotype distribution of rs605143 and rs565470 between CAD patients and control subjects. For rs605143, compared with women, for men, frequency of A allele is higher in control subjects than in CAD patients. There is no difference of A allele between CAD patients and control subjects in women. This result indicated that A allele of rs605143 is a protective factor for CAD in male patients. For total and men, the dominant model (GG vs AG + AA) was significantly higher in CAD patients than in control subjects, after multivariate adjustment of confounding factors such as plasma concentration of TG, TC, HDL, LDL, incidence of hypertension, diabetes and smoking for CAD, the significant difference was retained. This indicated that the G allele might be protecting against for CAD.

For rs565470, compared with women, for men, frequency of C allele is higher in CAD patients than in control subjects. There is no difference of C allele between CAD patients and control subjects in women. This result indicated that C allele of rs565470 is a risk factor for CAD in male patients. For total and men, the recessive model (CC vs TT + CT) was significantly higher in CAD patients than in control subjects, after multivariate adjustment of confounding factors such as plasma concentration of TG, TC, HDL, LDL, incidence of hypertension, diabetes and smoking for CAD, the significant difference was retained. This indicated that the C allele might be a risk factor for CAD.

In conclusion, polymorphisms of COL4A1 gene were associated with CAD in a Uygur population in China. Additional studies will need to be undertaken in order to clarify the underlying molecular mechanism which associates polymorphism of COL4A1 gene with CAD.

## Competing interests

All authors of this manuscript have declare that they have no competing interests.

## Authors’ contributions

Conceived and designed the experiments: DLR-AD, XX, Y-TM; Performed the experiments: DLR-AD, XX, B-DC; Analyzed the data: X-ML, FL, Z-YF, YX. Contributed reagents/materials/analysis tools: X-ML, Z-YF, FL. Wrote the paper: DLR-AD, XX, Y-TM, Y-NY. All authors read and approved the final manuscript.
